# Resection and Reconstruction for Lung and Airway Tumors Invading the Carina

**DOI:** 10.3390/cancers17020270

**Published:** 2025-01-15

**Authors:** Camilla Vanni, Erino A. Rendina, Giulio Maurizi, Antonio D’Andrilli

**Affiliations:** Division of Thoracic Surgery, Sant’Andrea Hospital, Sapienza University of Rome, 00185 Rome, Italy

**Keywords:** carinal resection, carinal pneumonectomy, carinal lobectomy, sleeve pneumonectomy, lung cancer, primary airway tumor

## Abstract

Tumors located at the tracheal bifurcation constitute a heterogeneous group of neoplasms whose treatment poses significant challenges due to their anatomical location, the requirement for radical resection, the need to restore local anatomy, and the necessity of maintaining adequate oxygenation throughout the entire procedure. Advances in airway reconstruction surgical techniques, anesthesia, and complementary therapies have progressively expanded indications for radical treatment of these neoplasms, resulting in significant improvements in both short- and long-term outcomes in recent years.

## Simple Summary

Carinal resection is a challenging thoracic surgical procedure indicated for the treatment of lung and primary airway tumors involving tracheobronchial bifurcation. This highly specialized operation combines tumor resection with airway reconstruction to restore anatomical continuity and preserve respiratory function. Advances in surgical techniques, perioperative management, and complementary therapies have significantly improved patient outcomes, making carinal resection a potentially curative option for carefully selected patients with otherwise inoperable disease. The procedure remains a pivotal strategy in thoracic oncology, requiring a multidisciplinary approach and the expertise of thoracic surgeons, anesthesiologists, and oncologists at high-volume centers.

## 1. Introduction

Tumors of the carina represent a heterogeneous group of neoplasms that pose significant technical challenges concerning the site of occurrence, the need to maintain adequate oxygenation, and the complexity of achieving complete resection. Surgical planning must consider the local extent of the disease, its aggressiveness, and the patient’s anatomical characteristics. In some cases, complex reconstructive procedures are required, with the aim of maximizing the preservation of healthy lung parenchyma.

Initial experiences of carinal resection principally concerned the treatment of rare, primary airway tumors with borderline malignancy that were minimally responsive to radiotherapy or chemotherapy and complex inflammatory strictures [1,2,3]. In contrast, non-small-cell lung cancer (NSCLC) primarily involving tracheobronchial bifurcation was largely considered inoperable due to the frequent local extension to vital adjacent structures and therefore managed palliatively with supportive care, endoscopic interventions, and chemo-radiotherapy protocols; until forty years ago, radical resection for T4-stage disease was reserved for exceptional cases with highly favorable anatomical and clinical conditions [4].

The early reports were focused on the feasibility, technical issues, and anesthesia management of carinal surgery and were often associated with high postoperative mortality and poor long-term oncologic survival rates [2,5]. Advancements in surgical techniques, anesthetic management, and complementary therapies (including both induction and adjuvant treatments), combined with the adoption of more rigorous patient selection criteria, have progressively contributed to improved outcomes over time [6,7].

Since the early 1990s, over 450 new papers on carinal resection have been published, a quarter of which were issued in the last five years. Nonetheless, the uncommon occurrence of cases eligible for carinal reconstruction, combined with the complexity of their management, have restricted the dissemination of technical expertise to high-volume centers with extensive experience and specialized skills in this field.

## 2. Indications for Surgery and Technique

In 1957, Barclay was the first to describe the complete resection of the tracheal carina with preservation of both lungs in a case of recurrent adenoid cystic carcinoma, then named cylindroma [1]. He restored the anatomical continuity of the tracheobronchial bifurcation by performing an end-to-end anastomosis between the right main bronchus and the tracheal stump, along with an end-to-side anastomosis of the left main bronchus to the intermediate bronchus. Due to the successful outcome of his case and the technical and anesthetic advancements introduced by his work, in subsequent years, other authors [2,3] published results from their pioneering experiences with circumferential tracheal-sleeve resection, with or without pneumonectomy, thus confirming the feasibility of these procedures and establishing the technical and management principles that are still in use today.

Reconstructive surgery of the carina is mostly oncological. Common indications are NSCLC that ascends to the tracheal bifurcation involving the origin of the main bronchus, the tracheobronchial angle or the carina itself, and primary airway neoplasms of the very distal portion of the trachea or the proximal bronchi, the more frequent of which are carcinoid, adenoid cystic carcinoma, and mucoepidermoid carcinoma. Less common indications also include isolated recurrence at the bronchial stump following pneumonectomy for lung cancer, complex benign strictures, and solitary metastatic lesions.

### 2.1. Considerations for Resection and Reconstruction

The indication for carinal reconstruction is based on a radical intent with the aim of preserving lung parenchyma whenever possible.

The first issue for the thoracic surgeon is to assess and define the resectability of the mass based on imaging, endoscopic, and intraoperative findings. The success of the procedure depends on achieving a tension-free, properly aligned anastomosis with precise apposition of viable, well-vascularized stumps. During dissection, it is therefore crucial to preserve the blood supply to the trachea provided by the segmental arteries and from the tracheal branches of the bronchial arteries and to minimize the skeletonization of the distal bronchial stumps [8]; direct electrocauterization of the airway must be strictly avoided, and, more generally, its use should be carefully managed to prevent both acute and delayed thermal injuries.

The resection can involve the carina alone or together with the right upper lobe (carinal lobectomy or tracheal-sleeve lobectomy), the right lung, or, less frequently, the left lung (carinal pneumonectomy or tracheal-sleeve pneumonectomy). The completeness of resection should be confirmed through intraoperative frozen section analysis of the tracheal and bronchial margins, guiding further resection if necessary. However, extending the length of resection may increase the tensile stress at the suture line, potentially leading to healing complications. If there are concerns about excessive tension at the anastomosis, in the presence of favorable histological subtypes, it is acceptable to limit the extent of resection and plan for subsequent adjuvant radiotherapy [9].

**Figure 1:**
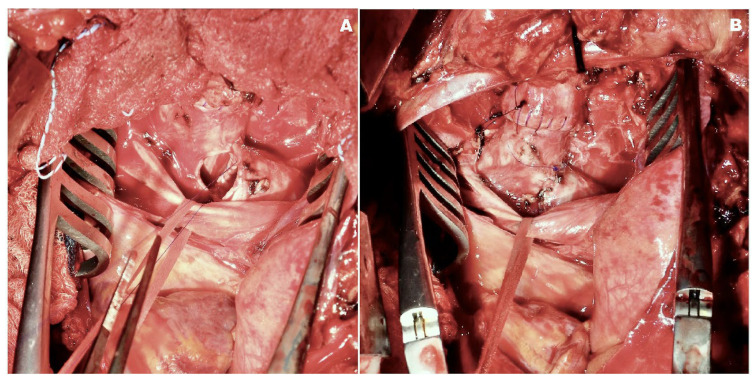
Intraoperative images demonstrating Barclay’s carinal reconstruction for mucoepidermoid carcinoma of the proximal left main bronchus. (**A**) The left main bronchus stump is sutured to the intermediate bronchus in an end-to-side manner. (**B**) Anatomical continuity is restored through both end-to-end and end-to-side anastomoses.

**Figure 2:**
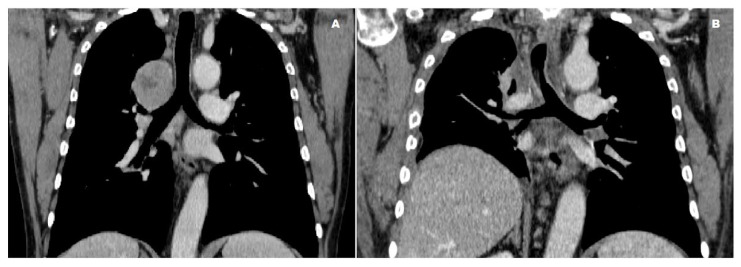
Preoperative (**A**) and postoperative (**B**) CT scans of a patient undergoing right carinal upper lobectomy for carcinoid tumor. (**A**) The preoperative scan reveals a tumor at the tracheobronchial angle without extension to the bronchus intermedius. (**B**) The postoperative scan shows the reconstructed bronchial tree with restored anatomy.

The available reconstructive options are varied and tailored to each case based on the disease’s location, its extent, and the individual’s anatomical features [10]. Reconstruction following carinal pneumonectomy involves direct anastomosis between the distal cut end of the trachea and the main bronchus of the remaining lung. If the resection includes the carina alone and its longitudinal extent is limited, the anatomy of the tracheobronchial bifurcation can be restored through the reconstruction of a neocarina [2]; this is generally performed by suturing together the medial aspect of the main bronchi and reanastomosing them to the distal trachea. Technical alternatives to neocarinal reconstruction have been described and employed to manage cases where the involvement and the subsequent length of resection of the main bronchi were asymmetric or where the tension between the bronchial stumps was disproportionate [1,3,10,11]. These variations included end-to-end approximation between the right main bronchus and the trachea with end-to-side anastomosis of the left main stump to an orifice created in the median wall of the intermediate bronchus (Barclay’s technique, Figure 1) or the distal trachea (Grillo’s technique) and, inversely, end-to-end anastomosis of the left mainstem to the trachea with end-to-side reimplantation of the right mainstem cranially to the tracheal anastomosis (Eschapasse’s technique) or to the median wall of the left main bronchus. This last is the same technique employed even after carinal right upper lobectomy, with the exception that is the intermediate bronchus to be end-to-side anastomosed (Figure 2).

Regardless of the nomenclature, a common challenge in all of these procedures is performing precise anastomotic sutures within a limited surgical field, often hindered by the great vessels of the mediastinum and the cross-field ventilation material. Consequently, the choice of the surgical approach is fundamental to achieve optimal exposure and must be defined on a case-by-case basis. Selection of the proper approach depends on the location of the neoplasm along with its biological aggressiveness and must consider the extent of proximal involvement of the trachea, the need for concomitant resection of the lung or release procedures, the suspicion of possible involvement of the mediastinal vessels, and, not least, the surgeon’s preference. Right-sided neoplasms are generally approached through a right thoracotomy, especially in the case of NSCLC or other primary lesions requiring concurrent lung resection; transthoracic incision of the fourth or fifth intercostal space offers optimal exposure of the carinal bifurcation and is routinely employed even in the case of isolated carinal resections. In a recent paper by Chen and coworkers, right posterolateral thoracotomy was employed as a choice in 91.7% of neocarinal reconstructions [12]. The right transthoracic approach allows for performing right hilar release, which makes it particularly suitable for elevating the intermediate bronchial stump in cases of carinal reconstruction and right upper sleeve lobectomy. However, the right-sided approach limits optimal exposure of the proximal airway and the ability to perform a left hilar release and should be excluded as an option whenever the resection of a long segment of trachea or the left main bronchus are expected. In contrast, a transpericardial approach through a median sternotomy offers the best exposure of the tracheobronchial bifurcation and is particularly indicated when the tumor straddles the trachea for a long section or when bilateral hilar and tracheal release procedures are needed. Median sternotomy is also the preferred method to approach left-sided lesions. Compared to the transthoracic approach, it offers the advantage of avoiding extensive preparation and retraction of the aortic arch to expose the tracheal plane, while also facilitating the setup of extracorporeal circulation when necessary [13]. Although less commonly used, left posterolateral thoracotomy is indicated in cases where a complex pulmonary resection is anticipated due to adhesions or when improved exposure of the posterior thoracic wall and the mediastinum is required due to local tumor extension; for complex, left-sided masses, combined approaches involving sternotomy and anterior thoracotomy have also been described [9]. In the last years, the use of minimally invasive video-assisted and robotic-assisted thoracic surgery (VATS, RATS) for tracheal and carinal resections have been discussed occasionally [14,15,16]. Although interesting, due to the limited number of experiences reported, the ideal setting, the highly selective type of patients treated, and the few surgeons who have attempted the procedure, VATS and RATS carinal surgery should be still considered anecdotical, and their feasibility needs further validation.

Given the anatomical location of the tracheal bifurcation within the middle mediastinum and its proximity to surrounding organs, a neoplasm at the carinal level, whether a primary carcinoma or the result of direct infiltration by lung cancer, may involve one or more surrounding structures. In the largest series of carinal surgeries, the incidence of concurrent en bloc resection of other mediastinal organs is up to 20%, which mostly involves vascular structures and is primarily correlated with NSCLC diagnosis [6,7,9,17]. The surgeon undertaking resection of the carina must assess the need to perform associated reconstructions and plan the procedure accordingly. Left-sided lesions are more often deemed unresectable due to the close proximity between the left main bronchus, the aorta, the pulmonary artery (PA), and the esophagus [18]. However, based on the authors’ previous experience, PA reconstruction has proven to be reliable after extended vascular sleeve resection, even in cases with widely separated PA stumps, by utilizing the interposition of a biological conduit [19]. On the right side, bronchogenic carcinomas cranially that involve the tracheobronchial angle frequently result in direct infiltration of the superior vena cava (SVC). Based on their extensive experience in carinal resection, de Perrot and coworkers reported the need for 25 SVC resections among a total of 96 patients who underwent right carinal pneumonectomy for bronchogenic carcinoma [7]. Complete SVC resection followed by prosthetic reconstruction (synthetic vascular graft) was performed in 13 cases, while partial resection of the vessel was carried out in 12. All of the procedures were performed through a right posterolateral thoracotomy at the fifth intercostal space. In contrast, the preferred approach at the Massachusetts General Hospital (MGH) for performing a right carinal pneumonectomy with en bloc caval resection was median sternotomy, which was combined with anterior right thoracotomy in 50% of cases [9].

### 2.2. Intraoperative Airway Management

Since the first reports on carinal surgery, the need to ensure adequate gas exchange throughout the entire resection and reconstruction process has represented one of the most challenging issues and required close cooperation between the thoracic surgeon and the anesthesiologist. The use of a thin, extra-long endotracheal tube, advanced into the bronchus to be reimplanted during the reconstruction phase, was the earliest described method [1,2,3]. This approach is still widely employed and is commonly combined with sterile cross-field ventilation during the resection phase of tracheal-sleeve pneumonectomy procedures [18,20,21,22]. While effective, this technique poses challenges, including partial obstruction of the tracheobronchial lumen during the anastomosis, which can complicate suturing. This issue is often mitigated by intermittent apnea during the placement of complex sutures. Nevertheless, in the case of carinal resection and carinal lobectomy, the often-inadequate gas exchange gained through the use of conventional ventilation material and, on the other hand, the discomfort and the limited space available for performing the end-to-side anastomosis with a ventilated lung have led to considering new solutions.

High-frequency jet-ventilation (HFJV) has emerged as a viable alternative to the traditional extra-long tracheobronchial tube and is increasingly adopted in both tracheal and bronchial reconstructive procedures [7,17,23,24,25,26]. From an anesthesiologic perspective, HFJV offers the significant advantage of maintaining adequate and consistent gas exchange during critical phases of reconstruction, eliminating the need for extended periods of intermittent apnea. From a technical standpoint, the reduced endoluminal footprint of the HFJV catheter minimizes interference within the surgical field, facilitating instrument maneuverability and anastomotic suturing. Additionally, the absence of obstructive ventilation materials allows for more precise work in confined surgical spaces, making HFJV particularly advantageous during minimally invasive thoracic approaches and the anastomotic phase with an inflated lung [27]. However, HFJV is not without potential complications; barotrauma may occur due to the high ventilation pressures, especially in cases where airway compliance is reduced. Additionally, HFJV requires meticulous anesthetic monitoring and advanced equipment, which may not be universally available. Improperly optimized jet settings can lead to hypercapnia or hypoxemia due to insufficient ventilation. Mucosal injury or airway trauma caused by the jet catheter, as well as difficulties in managing secretions, can further complicate the procedure.

The use of extracorporeal support during carinal surgery has been reported occasionally and is restricted to situations where complex dissection due to tumor proximity to the mediastinal vessels or complex exposure of the carinal and subcarinal region with the need for cardiac and vessel retraction are expected. Extracorporeal lung support is also privileged in situations where single lung ventilation is not sufficient to ensure adequate gas exchange due to anatomical or functional conditions or when severe endotracheal stenosis from bulky disease contraindicates or prevents the use of conventional intubation. Its application in selected cases has resulted in improved stability of patient oxygenation and enhanced comfort for the surgeon, thus increasing surgical precision and even reducing the reconstruction time [28]. Both veno-arterial and veno-venous extracorporeal membrane oxygenation (ECMO) and cardiopulmonary by-pass (CPB) have been employed in carinal neoplasms depending on the circumstances [9,22,29,30]. In the series from the cardiothoracic surgery of MGH [9], mechanical support was planned electively in four out of nine patients undergoing left carinal pneumonectomy; CPB was used in three patients because of tumor extension with suspected invasion of the aorta and the pulmonary artery or complex airway involvement requiring airway and cardiac mobilization and retraction, while veno-arterial ECMO was employed to assure adequate oxygenation in one patient with a very short right mainstem bronchus due to the concern about the effectiveness of right lung ventilation. In contrast, none of the 15 right-sided carinal resections or the 21 isolated neocarinal reconstructions reported in their series required extracorporeal support, thus confirming the additional technical challenges of left carinal pneumonectomy, which are primarily due to the close anatomical relationships between the left main bronchus, the aorta, the pulmonary artery, and the esophagus, which impair dissection and exposure of the surgical field.

From a technical standpoint, thoracic surgeons advocating for the use of ECMO over CPB during oncological surgery emphasize its lower incidence of related complications, including thromboembolic events and immunosuppression, as well as its reduced risk of tumor dissemination [28], which could affect both short- and long-term outcomes and access to adjuvant therapies. In the current authors’ previous series [22,31], no early or late related complications were observed, nor was there any impact on early recurrence in patients who received extracorporeal support during cardiovascular and airway reconstructive surgery for cancer.

Recent progress in anesthetic techniques has made it possible to perform airway reconstruction procedures without the need for endotracheal intubation. In the field of carinal surgery, published cases, although limited in number, have demonstrated the feasibility of this technique in appropriately equipped settings [16,32]. Potential advantages include the absence of airway obstruction and improved visualization of the surgical field during anastomotic suturing, the elimination of risks associated with intubation-related iatrogenic injuries and cuff-induced ischemic damage, and better postoperative recovery times. These range from the requirement for specialized training of both surgical and anesthetic teams to stringent eligibility criteria for the procedure that make it hardly applicable. The necessity of conversion to intubation due to intraoperative findings, alongside the need for prompt management of vascular complications within a precarious anesthetic setting, also highlight the complexity of this approach. For these reasons, the use of non-intubated surgery in carinal resection is still limited to sporadic cases in centers with adequate expertise in this field.

## 3. Operative Outcomes

Postoperative mortality following carinal resection procedures has progressively decreased over time, driven by advancements in surgical and anesthetic techniques, improvements in postoperative care, and more rigorous patient selection criteria. Over the past 40 years, mortality rates have more than halved, dropping from up to 40% in the 1980s and the 1990s [2,3,4,5,33] to current values of 3–10% in larger series [6,7,9,10,12,17,18,20,21,22,23,24,25,34,35,36,37] (Table 1).

A meta-analysis published by the American College of Chest Physicians in 2007 [38] highlighted that the highest long-term survival rates and the lowest perioperative mortality rates for patients undergoing carinal resection for T4 non-small-cell lung cancer (NSCLC) were reported in high-volume, experienced centers.

The operative outcome also appears to improve over time within the same center, in parallel with the increasing experience of the surgeon in performing and managing complex tracheobronchial reconstruction procedures. In their retrospective analysis of 60 cases of carinal resection performed over 25 years, Mitchell and colleagues observed a significant reduction in mortality rates, with a decrease of 50% between the first and second halves of their series [34].

In general, the incidence of operative mortality and postoperative complications following carinal resection are higher than those reported for standard major lung resections in the literature and seem to be influenced by the volume of parenchyma resected. A prominent series by de Perrot et al. [7] showed that postoperative mortality is more closely correlated with the type of lung resection than with the technical complexity of the reconstruction itself; notably, no mortality was observed by the authors following technically demanding procedures, such as carinal resection with right upper sleeve lobectomy, while the highest mortality rate (8.3%) was recorded in patients undergoing concurrent right pneumonectomy. Findings from another large French study on NSCLC involving the carina corroborated these results [17], reporting more favorable outcomes with isolated carinal resections compared to complete tracheal-sleeve pneumonectomy. Specifically, the morbidity rate after isolated carinal resection was 20%, with no reported mortality, whereas tracheal-sleeve pneumonectomy was associated with a morbidity rate of 54% and a mortality rate of 8%; in all cases, deaths were attributed to pulmonary complications.

Lung failure due to acute respiratory distress syndrome (ARDS) or postoperative pulmonary edema following tracheal-sleeve pneumonectomy has an incidence ranging between 5% and 15% in the largest series [7,17,23,34]. The occurrence of this complication is challenging to predict and is fatal in up to 80% of cases. Meticulous postoperative management focused on fluid balance and restricted fluid intake is considered crucial for the prevention of postoperative pulmonary edema, even though such measures may not always be sufficient to prevent the onset of non-cardiogenic ARDS. It has been hypothesized that the marked pulmonary congestion observed in some patients 24 to 72 h after carinal pneumonectomy may be attributed to the transection of nerves or the mediastinal lymphatic system, resulting in an impaired epithelial clearance mechanism. However, patients undergoing isolated carinal resection or carinal right upper lobectomy typically do not exhibit signs of respiratory distress. Sezen and colleagues recently published their results from a series of 64 patients undergoing carinal pneumonectomy (51) or lobectomy (13) [21]; the authors found that the incidence of ARDS was slightly higher in the carinal pneumonectomy group compared to the carinal lobectomy group (11.8% vs 7.7%) and was not associated with the extent of lymphadenectomy. Other authors reported no incidence of ARDS or pulmonary edema following lung-sparing carinal resection procedures in their series [17,34].

Airway complications range from granulation tissue formation to life-threatening events. Ischemic injury at the anastomosis can lead to abnormal healing, resulting in stenosis, or progress to dehiscence, potentially causing severe complications, such as bronchopleural or bronchovascular fistulas and complete anastomotic disruption. Dehiscence of the anastomotic sutures following circumferential carinal resection is a factor significantly affecting the perioperative outcome [39]. Mortality in patients developing an anastomotic fistula is higher after pneumonectomy compared to lung-sparing procedures and is greater in patients undergoing reoperation compared to those managed endoscopically. In the previously mentioned experience by Sezen and coworkers [21], five of the six patients who developed a bronchopleural fistula belonged to the pneumonectomy group and, although reoperation was attempted, the event was fatal in all of the five cases; in contrast, one case of fistula developed by a patient undergoing carinal lobectomy was resolved conservatively.

### Intra- and Perioperative Prevention of Complications

Careful and thorough postoperative monitoring and surveillance bronchoscopy are crucial to promptly detect any clinical change that may indicate the development of anastomotic complications. Early identification of a healing defect at the reconstruction site expands the possible treatment solutions. The immediate cessation of corticosteroids, the administration of hyperbaric therapy, and the use of a targeted endoscopic procedure with stent placement to cover the suture can halt the progression of dehiscence and reverse the outcome without the need for reoperation. Comparing outcomes from the initial [34] and the most recent experience [9] published by the MGH, the postoperative incidence of anastomotic complications has not only decreased from 17% to 11% but the mortality associated with these complications (previously reported to be 7% in the late postoperative period) has been reduced to zero through the effective use of endoscopic treatments and hyperbaric therapy. In the present authors’ experience, we adopt a conservative approach to managing anastomotic complications, favoring the use of endoscopic treatments, such as dilation and/or stenting, in cases of both stenosis and dehiscence. In particular, we have found that the early endoscopic exclusion of a fistula using self-expanding stents allows for rapid resolution of air leakage and significantly reduces the risk of progression to life-threatening complications, such as empyema and vascular erosion [22,40].

Excessive tension on anastomotic sutures caused by incomplete lung re-expansion or extensive length of the airway resected, along with reduced local trophism due to tumor effects or previous induction therapies, all represent factors affecting anastomotic healing [34]. Since the earliest reports of carinal resections, thoracic surgeons have employed various strategies to minimize traction on the anastomosis. These have included intraoperative release procedures (tracheal and intrapericardial hilar release) as well as the induction of pneumoperitoneum in the postoperative period [1,3]. The precautionary transposition of a vital tissue flap over the tracheobronchial sutures is also recommended by most authors [9,10,12,22,23]. This flap serves as a mechanical barrier against dehiscence and protects against fretting corrosion of the major mediastinal vessels and complications in the pleural cavity. Various tissues, including thoracic muscles, the parietal pleural flap, the pericardial fat pad, the thymic tissue, and the omentum, can be used depending on the circumstances. In the current authors’ practice, when approaching via thoracotomy, we routinely prepare the intercostal muscle flap from the selected intercostal space. In contrast, when a transsternal approach is planned, we consider the pericardial fat pad and the thymic tissue as the most readily accessible tissues [22].

## 4. Late Outcomes

Overall, long-term results after carinal surgery for cancer are quite variable due to the level of histological heterogeneity included in the study, the pathologic stage at the time of surgery, and the use of any multimodal treatment protocol.

Evidence from studies including various histologic subtypes suggests better long-term survival outcomes for patients undergoing carinal surgery for primary airway and neuroendocrine tumors compared to those with bronchogenic carcinomas. In the study by de Perrot et al. involving 119 patients [7], the 5- and 10-year survival rates for non-bronchogenic carcinomas were reported as 66% and 48%, respectively, compared to 44% and 25% for patients with an NSCLC diagnosis. Among the more favorable histologic subtypes, adenoid cystic carcinoma demonstrated a 10-year survival rate of 69%, while carcinoid tumors showed a survival rate of 100%. In contrast, less favorable outcomes were observed in patients with mucoepidermoid carcinoma and metastatic renal cell carcinoma within the non-bronchogenic group. These findings align with those reported in more recent studies. Costantino et al. [9] observed 5-year survival rates of 62% in patients with adenoid cystic carcinoma and 42% in those with bronchogenic carcinoma, demonstrating a notable median survival advantage for adenoid cystic carcinoma (6.4 years vs. 2.8 years). Similarly, the University of Padua group [41] reported significantly better long-term outcomes for adenoid cystic carcinoma compared to squamous cell carcinoma and adenocarcinoma, as evidenced by the univariate survival analysis by histology (*p* = 035). The data recently published by the current authors support these findings even in terms of disease-free survival [22]. In our retrospective series of 41 cases, we observed a recurrence rate of 11.1% for airway tumors compared to 41.3% for NSCLC patients, with a median follow-up of 29 months. Correspondingly, five-year disease-free survival rates were 85.7% for airway tumors and 48.3% for bronchogenic tumors.

Mediastinal lymph node metastases are well-established as the foremost independent negative prognostic factor affecting long-term survival in lung cancer [42]. In the context of carinal resection, a review of 5-year survival rates reported by various authors over the past 30 years revealed that the poorest outcomes are consistently associated with a higher prevalence of pathologic N2 disease (Table 2). An adequate preoperative assessment of lymph node involvement, with a particular focus on the mediastinal stations, is therefore crucial for determining the suitability of upfront surgery and has significant implications for long-term survival results [43]. Studies analyzing oncological outcomes in relation to nodal status have revealed a direct correlation between the extent of metastatic lymph node involvement and long-term survival, even in patients undergoing carinal resection [36,44]. In a series of 64 consecutive patients who underwent sleeve pneumonectomy at the thoracic surgery unit of Heidelberg University [25], the actual survival rates at 3 and 5 years were 40% and 31%, respectively. These outcomes were found to be strongly correlated with the pN stage, with 5-year survival rates of 70% for pN0 patients, 35% for pN1 patients, and 9% for pN2 patients. Similar findings have been reported by other authors over the years, including Mitchell [34], Yamamoto [36], and Rea [24], whereas de Perrot and colleagues [7] did not find significant survival differences at 5 years between patients with pN0 and pN1 disease (50% vs. 55%, respectively; *p* = 9).

Some authors have also identified the completeness of resection as an independent factor influencing long-term survival following circumferential resection of the carina for NSCLC [12,21,25]. Incomplete resection (R1 or R2) is generally associated with worse long-term results; however, extending the length of resection can increase anastomotic tension and lead to airway healing complications, potentially affecting immediate postoperative outcomes. While it is true that in the presence of T4 bronchogenic carcinoma, surgical treatment should be considered only for patients in whom radical tumor resection is achievable, further consideration is necessary for those with favorable histologic subtypes. Costantino and coworkers [9] found no significant difference in 5-year survival rates between patients with adenoid cystic carcinoma who underwent incomplete resections (62%, including both R1 and R2 resections) and those who underwent complete (R0) resection across all histologies (60%). In this subset of patients with incompletely resected adenoid cystic carcinoma, the best survival outcomes were achieved when adjuvant radiation therapy was employed.

As long as the resection margins are free of disease, local recurrence rates after carinal resection are low and similar to those achieved through standard pneumonectomy or bronchial sleeve procedures [12,20]. Moreover, the type of reconstruction, whether associated with lobar or complete lung resection or not, seems not to affect the risk of recurrence in the long term [22]. Roviaro and colleagues [18] reported five local recurrences and no recurrence at the anastomotic level in a total of 53 highly selected patients from 6 to 54 months after carinal pneumonectomy for NSCLC; at the follow-up of 23–97 months, 56.6% of patients were free from recurrence.

## 5. Induction Treatments

The safety and oncological worth of induction treatments in the field of thoracic surgery for NSCLC have been widely validated in the long term [45,46]. Neoadjuvant therapy, including concurrent chemoradiation, has been shown not to increase the risk of anastomotic-related complications following bronchial and vascular sleeve resections [47,48]. However, the available evidence on postoperative outcomes and long-term survival in patients undergoing carinal resections after induction regimens remains limited. Among the studies, those with a sufficient number of cases are difficult to compare due to different methodologies of treatment adopted (radiation, chemoradiation therapy, or chemotherapy alone) and the protocols used, including different inclusion, staging, and restaging criteria and preferences regarding surgical approaches and techniques.

The use of radiotherapy in induction protocols for T4 bronchogenic carcinoma involving the carina has been reported occasionally. In those patients receiving preoperative radiotherapy at doses ≥48 Gray, the incidence of anastomotic complications is higher, and long-term oncologic results are similar to or worse than those reported after neoadjuvant chemotherapy alone, with even high rates (61%) of no pathologic response to therapy [23,35].

While doubt persists regarding the effect of radiation therapy on tissue viability and anastomotic healing [39], most authors with significant experience in carinal reconstruction no longer consider neoadjuvant chemotherapy a limiting factor for surgery. The percentage of patients with NSCLC who received induction treatments before undergoing tracheal sleeve resection and reconstruction ranges between 14% and 36% in the major published series (Table 3). The indication to administer or not administer chemotherapy prior to surgery becomes increasingly critical in a context where the incidence of failure to receive recommended adjuvant therapies and treatment abandonment is high, particularly among patients undergoing pneumonectomy. It is well-known that that pneumonectomy is burdened by general and cardiac postoperative decline that may even preclude access to further therapies, thus jeopardizing the expected benefit from the completion of the multimodal treatment protocol. However, the line between tumor response to therapy and local damage to tissue tropism following induction therapy is narrow. An experimental study by Yamamoto et al. examined the correlation between preoperative and postoperative changes in bronchial perfusion and the healing of bronchial sutures [49]. The study documented a reduction in bronchial mucosal blood flow during surgery and impaired postoperative blood flow compensation through arteriolar communications in the bronchial wall in patients who received preoperative chemoradiation compared to those who received chemotherapy alone.

In general, the indication for preoperative treatments should be weighted while keeping in mind the current recommendation to recruit for induction the lymph node positive disease rather than the locally advanced (T3-T4 N0) resectable disease [50]. A study published in 2018 by Galetta and coworkers on this topic appears to support this recommendation [37]. The authors compared operative and long-term results of 22 patients who underwent carinal pneumonectomy after platinum-based chemotherapy for T4N2 NSCLC staged at presentation with those of 10 patients who received an upfront carinal pneumonectomy in the same period for T3 (less than 2 cm from the carina) N0-N1 NSCLC. The surgical results obtained in both groups were similar, with comparable rates of 30-day mortality (9.1%, n = 2 in the induction therapy group; 10%, n = 1 in the no-induction therapy group; *p* = 43), 90-day mortality (40.9%, n = 9 in the induction therapy group; 40%, n = 4 in the no-induction therapy group; *p* = 62), and morbidity (59.1%, n = 13 in the induction therapy group; 70%, n = 7 in the no-induction therapy group; *p* = 58). In particular, no difference in terms of bronchopleural fistula occurrence was noted. With regard to long-term survival, the authors reported a 5-year overall survival rate of 15.6% in the group of patients with T4N2 lung cancer who received neoadjuvant therapy compared to 60% in the group with stage T3N0-1 disease who underwent upfront surgery, although the recurrence rate was similar in the two groups (31.8%, n = 7 in the post-induction group; 30%, n = 3 in the no-induction group; *p* = 62). The discordant outcomes appear problematic to compare due to the different local and nodal stages at presentation; however, it is noteworthy that among the 13 patients who had downstaging following induction treatment, the recorded 5-year survival rate was 42.9%. A similar advantage was observed by Collaud et al. [51], who reported that pathological response to neoadjuvant treatments provided a significant prognostic benefit in 5-year outcomes in patients with resected T4 NSCLC. Specifically, survival rates were 86% in patients with pathological downstaging (ypT0-3) compared to 19% in non-responders (ypT4). These findings are primarily related to conventional neoadjuvant chemotherapy and chemoradiotherapy regimens. However, additional considerations will need to be addressed in the near future, given the emerging role of immunotherapy in induction protocols for stage III NSCLC [52].

## 6. Conclusions

Although technically challenging, carinal resection represents the treatment of choice for tumors localized to the tracheobronchial bifurcation, whether of NSCLC histology or primary airway origin. The trend toward favoring lung-sparing procedures when indicated, along with increased expertise in the early recognition and management of airway complications, have significantly reduced operative mortality, which remains high in patients who develop respiratory distress. Long-term outcomes in appropriately selected cases are satisfactory, even after the administration of neoadjuvant therapy. The potential impact of immunotherapy on the management and survival of these patients needs to be addressed in future studies.

Carinal resection represents the treatment of choice for most tumors localized to the tracheobronchial bifurcation, whether of NSCLC histology or primary airway origin. Given the rarity of these conditions and the challenges in their management, referral to high-volume specialized centers for comprehensive multidisciplinary evaluation and treatment is strongly recommended, allowing for satisfactory outcomes in both the short and long term. The trend toward favoring lung-sparing procedures, when indicated, combined with advanced expertise in the early recognition and management of airway complications, have contributed to reducing operative mortality, which remains high in patients who develop respiratory distress. Long-term outcomes in appropriately selected cases are satisfactory even after the administration of neoadjuvant therapy. The potential role of immunotherapy in improving the management and survival of these patients needs to be addressed in future studies.

## Figures and Tables

**Table 1:** Published experiences of ≥30 carinal resections in the last 30 years.

Author	Pts	Morbidity	Operative Mortality
Dartevelle (1995) [6]	55	12.7% ^§^	10.9% °
Mitchell (2001) [34]	60	45%	15% ^†^
Porhanov (2002) [23]	231	35.4%	16%
Regnard (2005) [17]	65	50.8%	7.7%
de Perrot (2006) [7]	119	47.1%	7.6%
Roviaro (2006) [18]	53	11.3% *	7.5%
Macchiarini (2006) [35]	50	37%	4%
Yamamoto (2007) [36]	35	22.8% *	8.5%
Rea (2008) [24]	49	28.6%	6.1%
Jiang (2009) [10]	41	60.9%	2.4%
Eichhorn (2013) [25]	64	40.6%	3.1%
Shin (2014) [20]	30	36.7%	0%
Galetta (2018) [37]	32 ^^^	62.5%	9.4%
Sezen (2018) [21]	64	48.8%	10.9%
Costantino (2019) [9]	45	58% ^‡^	6.8%
Chen (2021) [12]	36	38.9%	5.6%
D’Andrilli (2024) [22]	41	24.3% *	7.3%

^§^ Only cases of empyema reported. ° The reported operative mortality was 0% in the second half of the study period. ^†^ The reported operative mortality was 20% in the first half of the series and 10% in the second half. * Major complications. ^^^ All post-induction therapy patients. ^‡^ Twelve patients experienced more than one complication.

**Table 2:** Overall and pN2-related long-term survival rates in the largest series on carinal surgery.

Author	NSCLC pts	pN2 (%)	Overall 5-y Survival Rate	pN2 5-y Survival Rate
Mitchell (1999) [34]	60	11 (18.3%) *	42%	12% *
Regnard (2005) [17]	65	23 (35.4%)	26.5%	5.3%
de Perrot (2006) [7]	100	27 (27%)	44%	15% *
Roviaro (2006) [18]	53	12 (22.6%)	33.4%	-
Macchiarini (2006) [35]	50	18 (36%)	51%	-
Yamamoto (2007) [36]	34	10 (29.4%)	28.3%	0%
Rea (2008) [24]	49	15 (30.6%)	27.5%	0%
Eichhorn (2013) [25]	64	26 (41%)	41%	9%
Sezen (2018) [21]	64	7 (10.9%)	42.2%	22.2% (2-year)

* Refers to both pN2 and pN3 patients.

**Table 3:** Number of patients receiving induction treatments in the largest series on carinal surgery.

Author	NSCLC pts	Post-Induction Therapy pts (%)
Mitchell (1999) [34]	60	9 (15%)
Porhanov (2002) [23]	157	33 (21%)
Regnard (2005) [17]	65	11 (17%)
de Perrot (2006) [7]	119	23 (19%)
Macchiarini (2006) [35]	50	18 (36%)
Rea (2008) [24]	49	19 (39.6%)
Eichhorn (2013) [25]	50	8 (16%)
Sezen (2018) [21]	51	9 (14%)
